# Collagen sealant patch to reduce lymphatic drainage after lymph node dissection

**DOI:** 10.1186/1477-7819-10-275

**Published:** 2012-12-19

**Authors:** Gianluca Di Monta, Corrado Caracò, Anna Crispo, Ugo Marone, Nicola Mozzillo

**Affiliations:** 1 Department of Surgery, “Melanoma, Soft Tissues, Head and Neck, Skin Cancers”, National Cancer Institute of Naples, Naples, 80131, Italy; 2 Department of Epidemiology, National Cancer Institute of Naples, Naples, 80131, Italy

**Keywords:** Melanoma, Lymph node dissection, Fibrin sealant

## Abstract

**Background:**

Seroma formation is a frequent complication following radical lymph node dissection (RLND) in patients with metastatic melanoma. Several strategies have been used to prevent fluid accumulation and thereby reduce the duration of postoperative drainage, including fibrin sealants.

**Methods:**

This was a prospective, single-center study in which consecutive patients undergoing surgical treatment of stage III metastatic melanoma by axillary or ilio-inguinal RLND were randomized to receive standard treatment plus fibrinogen/thrombin-coated collagen sealant patch (CSP) or standard treatment alone. The primary endpoint of the study was postoperative duration of drainage.

**Results:**

A total of 70 patients underwent axillary (n = 47) or ilio-inguinal (n = 23) RLND and received CSP plus standard treatment (n = 37) or standard treatment alone (n = 33). Mean duration of drainage was significantly reduced in the CSP group compared with standard treatment (ITT analysis: 20.1 ± 5.1 versus 23.3 ± 5.1 days; p = 0.010). The percentage of patients drainage-free on day 21 was significantly higher in the CSP group compared with the standard treatment group (86% versus 67%; p = 0.049).

**Conclusions:**

Use of the tissue sealant resulted in a significant reduction in duration of drainage. Further studies are warranted to confirm these results in different and selected types of lymphadenectomy.

## Background

Radical lymph node dissection (RLND) is generally proposed as the only treatment for local control of node-positive stage III melanoma patients. However, ilio-inguinal and axillary dissections have been associated with a number of postoperative complications, ranging from wound infections to skin flap necrosis. These can result in increased hospital costs, reduced quality of life of patients and a delayed return to work and other activities. The incidence of complications after ilio-inguinal and axillary lymphadenectomy varies greatly. In previous studies, axillary RLND in patients with melanoma resulted in a local complication rate of between 25% and 48%, with over half of the observed complications being delayed wound healing secondary to serum collection [1,2]. Similarly, postoperative complications related to seroma formation occurred in 32% to 80% of patients after ilio-inguinal RLND [3,4].

Intraoperative insertion of closed suction drains at the time of RLND is the traditional method used to reduce seroma formation and subsequent wound complications. However, closed suction drains require ongoing surveillance and maintenance, restrict patients’ physical activity and delay their return to normal life. They can also be associated with complications such as infection or flap necrosis. Consequently, effective strategies to reduce the incidence of seroma formation and thereby allow earlier drain removal offer benefits to both patients and surgeons.

A variety of different methods to reduce seroma formation have been previously proposed, all of which aim to reduce dead space and seal leaking capillaries and lymphatic channels. These approaches include closing dead space by tacking sutures [5], delayed exercise [6,7], spraying tetracycline [8], using ultrasound cutting devices [9], applying bovine thrombin during surgery [10], and using fibrin sealants [11,12]. This latter approach has been proposed to provide sealing and tissue apposition in a large number of surgical procedures, especially after surgery for breast cancer, although results have been mixed [4,13-16].

One strategy may be the use of a fixed combination of collagen matrix-bound coagulation factors. TachoSil^®^ (Nycomed, Takeda Pharmaceuticals International GmbH, Zurich, Switzerland) is a tissue sealant consisting of an equine collagen sealant patch (CSP) coated with human fibrinogen and thrombin. It is ready to use, and differs from other sealing agents in being a fixed patch that is activated by the operated surface after 3 to 5 minutes of compression [17]. The efficacy and safety of CSP has been shown in several different surgical fields including hemostasis and bile sealing in liver surgery [18,19], air sealing in pulmonary lobectomy [20-22], and hemostasis in kidney tumor resection [23], cardiovascular and vascular surgery [24,25]. CSP is approved worldwide and is indicated in adults for supportive treatment in surgery for improvement of hemostasis, to promote tissue sealing, and for suture support in vascular surgery where standard techniques are insufficient.

In this study, the use of CSP to decrease seroma formation and reduce the time to closed suction drain removal was investigated in patients with metastatic melanoma undergoing RLND.

## Methods

This was a prospective study carried out at a single center in which consecutive patients were randomized to receive standard treatment plus CSP or standard treatment alone. The study was conducted according to the Declaration of Helsinki and in accordance with Good Clinical Practice. All patients provided written informed consent.

Patients were eligible for the study if they were candidates for surgical treatment of metastatic melanoma stage III by axillary or ilio-inguinal RLND. Previous radiation therapy at the operative site was an exclusion criterion. Patients were also excluded if they were obese with a body mass index (BMI) > 30 kg/m^2^, were pregnant or lactating, had received steroids within the past 6 months, or had a severe pre-existing concomitant medical condition. Randomization was achieved by drawing lots.

### Surgical technique

All surgery was performed by the Head of Department or by a senior member of his team. Axillary dissection was carried out through a transverse/S-shaped incision, raising 1 cm thick skin flaps by electrocautery to the lateral border of the pectoralis major and latissimus dorsi muscles. RLND was realized on level I, II and III, *en bloc* with the pectoralis minor muscle and supra-axillary fat pad, preserving the thoracodorsal neurovascular bundle and the long thoracic nerve. Major vessels, but not lymphatic vessels, were ligated, while fat dissection was carried out by electrocautery. At the end of surgery, a closed suction drain was inserted through the inferior skin flap and into the axilla.

Inguinal RLND was initiated with a lazy S incision extending from the anterior superior iliac spine to the apex of the femoral triangle. Skin flaps were raised using electrocautery medially to the pubic tubercle and to the adductor longus muscle, and laterally to the apex of the femoral triangle. The fatty, node-bearing tissue of the triangle was removed, skeletonising the femoral bundle in a subadventitial plane and transecting the saphenous vein at the confluence. Access to the iliac fossa was obtained via a transverse incision through the external oblique aponeurosis, 4 cm above the inguinal ligament, lifting up the peritoneum. RLND was performed around the external iliac vessels up to the level of the ureter and down in the pelvis to the obturator nerve. Finally, the muscular and aponeurosis edges were sutured, and the wound closed over a closed suction drain in the femoral triangle.

In the CSP group, the patches were applied subcutaneously to cover the whole surgical bed before closure after RLND.

### Postoperative care

All patients were instructed to reduce physical activity for the first week, to shower regularly, to record daily drainage volume and to return to the hospital for weekly follow-up visits to a dedicated outpatient nurse team, blinded to the received treatment. Drainage was removed when the 24-hour drainage volume had decreased to less than 50 cc for two consecutive days (in which case patients were instructed to return to the hospital the following day) or 30 days after surgery.

### Statistical analysis

The primary endpoint of the study was post-operative duration of drainage. Secondary endpoints were drainage removal incidence and daily drainage volume on post-operative days 3, 10, 15 and 21. Intention-to-treat (ITT) analysis was used for all endpoints. Per-protocol (PP) analysis was also conducted for the primary endpoint.

All statistical analyses were carried out with the SAS^®^ software package (SAS, Version 9.2, SAS Institute Inc., Cary NC, USA). *P*-values were considered significant if < 0.05. Categorical variables were compared using the chi square or Fisher exact test when appropriate. Continuous variables were compared using the unpaired *t*-test or the non-parametric Wilcoxon’s independent sample test according to whether or not the data distribution was normal as evaluated by the D’Agostino-Pearson test. The probability of drainage removal was also assessed using Kaplan-Meier curves and a log-rank test used to compare groups.

## Results

Between July 2009 and November 2010, 70 patients with melanoma underwent an axillary or ilio-inguinal RLND and were included in the study. Of these patients, 47 had axillary metastases and 23 had inguinal metastases. Thirty-seven patients were randomized to the standard treatment plus CSP group (axillary metastases, n = 26; inguinal metastases, n = 11) and 33 were randomized to the control group receiving standard treatment only (axillary metastases, n = 21; inguinal metastases, n = 12).

In the group with axillary disease treated with CSP, 6/26 patients (23%) underwent dissection after sentinel node biopsy (SNB), while in the standard treatment group 6/21 (28%) underwent dissection for clinical disease. In the group with groin disease treated with CSP, 1/11 patients (9.1%) were submitted to dissection after SNB, while 2/12 (16.6%) in the standard treatment group were submitted to dissection for clinical disease. Surgical procedures after positive SNB and after palpable disease were identical.

All 70 patients were included in the ITT analysis. Two patients in the standard care group had their drains removed because of complications (dehiscence) and so were excluded from the PP analysis.

The two treatment groups were comparable with regard to age, sex, number of removed lymph nodes and number of positive lymph nodes (Table 1). The median age of the patients was 59 years (range 29 to 82) in the CSP group and 64 years (26 to 84) in the standard treatment group (*P* = 0.65). The median number of lymph nodes removed was 20 (14 to 33) in the CSP group and 19 (10 to 34) in the standard treatment group. In the TachoSil^®^ group, the median number of positive lymph nodes was 2 (0 to 13) while in the standard treatment group it was 1 (0 to 15). These values do not include the sentinel lymph node, which was surgically removed in all patients.

**Table 1:** Patient characteristics

	**TachoSil^®^ (n = 37)**	**Standard treatment (n = 33)**	***P*-value**
Sex, female/male	15/22	12/21	0.720
Age, years*	59 (29, 82)	64 (26, 84)	0.650
Body mass index, kg/m^2^*	19.5, 24.3	20.4, 24.3	-
Lymph nodes positive*	0, 13	0, 15	0.204
Lymph nodes removed*	14, 33	10, 34	0.800
Sentinel node cases, n	7	8	-
Clinical disease cases, n	30	25	-

*Data given as median and/or (range).

### Duration of drainage

Duration of drainage is reported in Table 2. In the ITT population, the mean duration of drainage was significantly reduced in the CSP group compared with standard treatment (20.1 ± 5.1 versus 23.3 ± 5.1 days; *P* = 0.010). This significant difference in drainage duration was also observed in the PP analysis, which excluded two standard treatment patients (20.1 ± 5.1 days in the CSP group versus 24.0 ± 4.6 days in the standard treatment group; *P* = 0.002). These findings are confirmed by the Kaplan-Meier analysis, in which drainage removal occurred significantly earlier in the TachoSil^®^ group compared with standard treatment. The result of the log-rank test for the comparison between the two curves is statistically significant in favor of the CSP group (*P* = 0.024).

**Table 2:** Mean ± SD duration of closed suction drain placement in patients treated with TachoSil^®^ versus standard treatment

	**TachoSil^®^ (n = 37)**	**Standard treatment (n = 33)**	***P*-value**
Day of drainage removal (ITT)	20 (5)	23 (5)	0.010
Day of drainage removal (PP)	20 (5)	24 (4)	0.002

*ITT* intention-to-treat; *PP* per protocol.

### Drainage removal incidence

The percentage of patients drainage-free on day 21 was significantly higher in the CSP group compared with the standard treatment group (86% versus 67%; *P* = 0.049) (Table 3). No statistically significant differences between groups were detected at the other time points assessed (days 3, 10 and 15).

**Table 3:** Cumulative incidence of drainage-free patients (%) treated with TachoSil^®^ versus standard treatment on postoperative days 3, 10, 15 and 21

**Postoperative day**	**TachoSil^®^ (n = 37)**	**Standard treatment (n = 33)**	***P*-value**
Day 3	0	0	-
Day 10	0	3	1.0
Day 15	22	9	0.133
Day 21	86	67	0.049

Results are presented as percentage of patients who were drainage-free.

### Drainage volume

Daily drainage volume did not significantly differ between the CSP group and the standard treatment group on days 3, 10 or 15, but approached statistical significance on day 21 (25.7 ± 89.5 versus 39.7 ± 63.9 cc; *P* = 0.058) (Table 4).

**Table 4:** Mean ± SD daily drainage volume in patients treated with TachoSil^®^ versus standard treatment on postoperative days 3, 10, 15 and 21

**Postoperative day**	**TachoSil^®^**	**Standard treatment**	***P*-value**
	**(n = 37)**	**(n = 33)**	
Day 3	175.8 ± 95.3	170.0 ± 59.4	0.844
Day 10	143.1 ± 128.1	162.1 ± 86.8	0.096
Day 15	96.1 ± 109.9	104.3 ± 71.5	0.214
Day 21	25.7 ± 89.5	39.7 ± 63.9	0.058

Results are presented as mean drainage volume ± SD (cc).

### Complications

There were no intraoperative complications in either group. Two patients in the standard treatment control group had a dehiscence after ileo-inguinal dissection; one because of partial skin flap necrosis and one because of wound infection. No other major postoperative complications occurred and there was no perioperative mortality.

## Discussion

The most common complication after RLND is fluid collection in the wound. The pathophysiology of seroma formation is not clearly understood and different hypotheses have been proposed, largely based on evidence from patients undergoing axillary RLND after mastectomy. Woodworth, *et al*. (2000) suggested that seroma formation is a consequence of surgical disruption of lymphatics and capillaries with ensuing leakage of fluid into the dead space created by surgical dissection [26]. Others have proposed that seroma is a consequence of inflammatory exudates, showing high concentrations of proteins in fluid aspirates [27]. Previous studies suggest that the bulk of the fluid accumulation after RLND is not simply lymph from divided lymphatic vessels. However, neither it is merely a collection of serum, but rather consists of fluid formed by acute inflammatory exudates in response to surgical trauma and the acute phase of wound healing [27,28].

Most surgeons use closed suction drainage following RLND, with the duration of drainage in melanoma patients differing widely, from 4 to 5 days in one report [29], to 26 to 31 days in another study [4]. Evidently, drainage duration largely depends on the surgical practice and routine of the particular surgeon or center and the results of individual trials must be considered in this context.

Postoperative wound drainage has a significant negative impact on patients’ well-being, interfering with their normal daily activities, reducing sleep and delaying their return to work. In a study of melanoma patients who had undergone inguino-femoral lymph node dissection, Mortenson and coworkers found that getting dressed, bathing, and sleeping was a major problem for 67%, 78% and 72% of patients, respectively [4]. The majority of these patients were theoretically willing to pay US$100 to US$200 out-of-pocket costs to reduce the time of drain removal by 4 days or more, indicating the significant impact drains have on patient’s quality of life [4].

The use of different surgical techniques, including avoidance of electrocautery, tacking sutures, ultrasound cutting devices, and modified lines of incision, have proven largely ineffective in preventing or reducing fluid collection after RLND [5-9]. The addition of fibrin sealants have also been disappointing in lymphadenectomy for melanoma [4,29,30]. In one randomized controlled trial of 30 melanoma patients, fibrin sealant did not decrease seroma output or time to drain removal following ilio-inguino lymph node dissection [4]. In two other randomized studies in which patients with malignant melanoma underwent either axillary (n = 58) or combined radical ilio-inguinal (n = 58) dissection, the intraoperative application of fibrin sealant did not reduce the duration of closed suction drainage or length of postoperative hospital stay [29,30].

Both of these previous studies were comparable to the present study with regard to number of patients, site of lymphadenectomy, and number of removed lymph nodes. However, in the current study the use of the tissue sealant resulted in a reduction in duration of drainage of approximately 3 to 4 days compared with standard treatment alone. Differences of this extent have previously been reported as meaningful for patients [4].

It is not clear why these results differ from previous studies of other sealants. However, CSP differs from the liquid fibrin sealants used in earlier studies, due to the presence of the collagen fleece which serves as additional layer for sealing. This may be one possible explanation for the benefits observed here. Results shown in this article are also consistent with those of a similar randomized prospective pilot trial of CSP to prevent seroma after pelvic lymphadenectomy for urologic malignancies [31]. In this, the use of TachoSil^®^ effectively reduced drainage volume and prevented lymphocele development after extraperitoneal radical retropubic prostatectomy for prostate cancer.

## Conclusion

Current study findings confirm and support previous suggestions that TachoSil^®^ is an effective tissue sealant that can reduce lymphatic leakage following surgery. Further studies are warranted to confirm these results in longer series and in different and selected types of lymphadenectomy.

## Competing interests

No conflict of interests to declare. TachoSil^®^ was provided by the hospital pharmacy free of charge.

## Authors’ contributions

NM conceived the study, drafted the manuscript and performed surgical procedures. GDM helped to draft the manuscript and carried out the literature research. CC and UM helped surgical procedures and management of the patients. AC performed statistical analysis. All authors read and approved the final manuscript.
